# Developing a decision support tool to predict delayed discharge from hospitals using machine learning

**DOI:** 10.1186/s12913-024-12195-2

**Published:** 2025-01-11

**Authors:** Mahsa Pahlevani, Enayat Rajabi, Majid Taghavi, Peter VanBerkel

**Affiliations:** 1 Department of Industrial Engineering, Dalhousie University, PO Box 15000, Halifax, B3H 4R2 NS Canada; 2 Management Science Department, Cape Breton University, 1250 Grand Lake Road, Sydney, B1M 1A2 NS Canada; 3 Sobey School of Business, Saint Mary’s University, 923 Robie St., Halifax, B3H 3C3 NS Canada

**Keywords:** Discharge prediction, ALC patients, Machine learning, Discharge planning, Delayed discharge

## Abstract

**Background:**

The growing demand for healthcare services challenges patient flow management in health systems. Alternative Level of Care (ALC) patients who no longer need acute care yet face discharge barriers contribute to prolonged stays and hospital overcrowding. Predicting these patients at admission allows for better resource planning, reducing bottlenecks, and improving flow. This study addresses three objectives: identifying likely ALC patients, key predictive features, and preparing guidelines for early ALC identification at admission.

**Methods:**

Data from Nova Scotia Health (2015-2022) covering patient demographics, diagnoses, and clinical information was extracted. Data preparation involved managing outliers, feature engineering, handling missing values, transforming categorical variables, and standardizing. Data imbalance was addressed using class weights, random oversampling, and the Synthetic Minority Over-Sampling Technique (SMOTE). Three ML classifiers, Random Forest (RF), Artificial Neural Network (ANN), and eXtreme Gradient Boosting (XGB), were tested to classify patients as ALC or not. Also, to ensure accurate ALC prediction at admission, only features available at that time were used in a separate model iteration.

**Results:**

Model performance was assessed using recall, F1-Score, and AUC metrics. The XGB model with SMOTE achieved the highest performance, with a recall of 0.95 and an AUC of 0.97, excelling in identifying ALC patients. The next best models were XGB with random oversampling and ANN with class weights. When limited to admission-only features, the XGB with SMOTE still performed well, achieving a recall of 0.91 and an AUC of 0.94, demonstrating its effectiveness in early ALC prediction. Additionally, the analysis identified diagnosis 1, patient age, and entry code as the top three predictors of ALC status.

**Conclusions:**

The results demonstrate the potential of ML models to predict ALC status at admission. The findings support real-time decision-making to improve patient flow and reduce hospital overcrowding. The ALC guideline groups patients first by diagnosis, then by age, and finally by entry code, categorizing prediction outcomes into three probability ranges: below 30%, 30-70%, and above 70%. This framework assesses whether ALC status can be accurately predicted at admission or during the patient’s stay before discharge.

## Introduction

The increasing demand for healthcare services has led to challenges in managing patient flow within the hospital system. This rising demand has several origins, including population growth, aging demographics, advancements in medical technology, and the prevalence of chronic diseases [1, 2]. As healthcare needs rise, hospitals must manage complex resource allocation, reduce wait times, and ensure timely access to care. Efficient patient flow is needed to minimize delays and reduce the risk of adverse events or complications [3].

By streamlining the flow of patients, hospitals can optimize their capacity to meet growing demand, leading to improved patient outcomes and satisfaction. Moreover, effective patient flow management contributes to healthcare systems’ overall efficiency and cost-effectiveness [3, 4].

With an aging population, there is an increased demand for Long Term Care (LTC) beds. Given LTC bed shortages, patients often experience extended hospital stays and delayed discharge. In Canada, this population of patients is designated as Alternative Level of Care (ALC) as they no longer need acute care, in contrast to the usual hospital patient [5]. ALC patients cannot be discharged to their homes or communities due to a lack of available resources or support outside the hospital, such as home care or community-based services [6].

Extended hospital stays beyond medical necessity can strain healthcare resources, leading to increased healthcare expenditures and inefficient use of limited hospital capacity [7–9]. Furthermore, delayed discharges affect the broader healthcare system and cause challenges related to capacity management, patient flow, and care coordination. These challenges include hospital overcrowding, prolonged wait times, and delays in providing care to those who need it the most [8, 10]. The cost implications associated with ALC patients also underscore the importance of addressing these issues effectively.

ALC patients face an increased risk of healthcare-associated adverse events due to hospital environments that are ill-suited to meet their specific needs. These findings underline the importance of raising awareness among stakeholders about the risks associated with ALC stays and advocating for strategies to minimize the duration of ALC days. Lim et al. [11] investigate healthcare-associated adverse events in ALC patients awaiting LTC in two hospitals. Results indicate significant adverse events in ALC patients, such as infections, antimicrobial days, and non-infectious adverse events.

Early identification of individuals at risk of being ALC can be crucial in optimizing patient flow and resource allocation [12]. Using predictive analytics, healthcare providers can identify potential ALC patients in advance, allowing them to anticipate the needs and resource requirements of these patients. This enables more effective planning, decision-making, and intervention opportunities [5, 8, 9]. Additionally, a potential solution to address the challenge of ALC patients involves optimizing capacity planning within community-based services. Predictions about the ALC status of patients in the hospital can assist in the effective management of healthcare services for ALC patients.

This study employs two phases with three Machine Learning (ML) models: Random Forest (RF), Artificial Neural Network (ANN), and eXtreme Gradient Boosting (XGB) to predict whether a patient will become ALC. Phase one utilizes all patient features, while phase two uses only admission-time features. The performance of each classification algorithm is evaluated using various metrics, including precision, recall (sensitivity), F1-score, Area Under the Curve (AUC), and specificity with a 95% confidence interval, to determine the best-performing model.

Moreover, SHapley Additive exPlanations (SHAP) analysis is conducted to identify the key features, enabling the development of a guideline based on the three most predictive features at admission. This guideline enables hospital staff to identify patients likely to become ALC at any point during their stay without needing computers or ML models. It also assists in making informed decisions when treating ALC patients, ultimately enhancing overall patient care within healthcare settings.

To our knowledge, this is the first study to predict factors related to ALC patients using a real and large dataset. This study also addresses analysis challenges, such as handling a big dataset that is highly imbalanced. Another contribution is that identifying primary patient features is critical for predicting ALC status at admission. These features form the basis for developing a guideline, functioning as a tool to predict patients likely to become ALC.

The rest of this paper is organized as follows. Literature review section provides a literature review of previous research on ALC patients and the use of ML in discharge prediction and identifies research gaps. Methodology section presents the methodology, including data description, data preprocessing steps, and the different ML models used in this study. In Results section, the performance of ML models is evaluated, a comprehensive analysis of the predictors is conducted, and a prediction guideline is created using the variables contributing to ALC status. Finally, Conclusion section concludes the study and outlines potential future research directions.

## Literature review

To examine the findings in this area and identify gaps in the literature, this section reviews studies on delayed discharge, ALC patients, and ML-based predictions for patient discharge. Additionally, a recent review study [13] offers a comprehensive understanding of the various methods used to predict patient outcomes, detailing key methodologies, significant findings, and their implications for discharge planning.

### ALC patients

Several studies investigate delayed discharge issues by focusing on patient characteristics and healthcare system factors that impact the probability of delayed discharge [14–16]. Research indicates that patient demographics and healthcare factors are critical in understanding delayed discharge patterns and ALC patient designation risks.

A consistent finding across studies is that patient demographics and diagnosis contribute significantly to LOS and delayed discharge risk. Victor et al. [17] investigate the primary factors independently contributing to delayed discharge, including the absence of a family carer, admission to a nursing home, and staffing levels of the discharge assessment team, while McClaran et al. [18] further demonstrate that characteristics such as marital and parental status correlate with prolonged LOS and ALC classification.

Using data from the Discharge Abstract Database (DAD) on patients with acquired brain injury, Chen et al. [7] find significant factors contributing to ALC days include having a psychiatric comorbidity, age, and a longer LOS in acute care. Similarly, the prevalence of dementia among ALC patients, as observed by McCloskey et al. [10], and Little [8], underscores the importance of early diagnosis and targeted community-based support to reduce the risk of extended hospital stays for these patients.

In a thesis by Ahmed (2019) [19], it is highlighted that urban residents with lower incomes and higher instability and dependence levels are more prone to ALC days. In a retrospective cohort study, Bai et al. [9] evaluate hospital costs and complications among general internal medicine inpatients with delayed discharge, establishing a clinical prediction rule to identify at-risk patients. Their results show that delayed discharge is associated with higher hospital expenses and increased complication rates, especially nosocomial infections.

Healthcare system factors influencing delayed discharge have also been studied. Cadel et al. [20] highlight the significance of consistent collaboration with physicians, active involvement of senior leadership in engaging frontline providers, and integrating the community sector into discharge planning.

Micallef et al. [21] found that delayed discharges are attributed to organizational mismanagement, insufficient planning, and care transfer difficulties. Consequences include bed-blocking, emergency department overcrowding, and financial implications. To address these issues, they suggest initiatives like the ’discharge before noon’ program, the use of ’discharge facilitation tools,’ the establishment of ’discharge delay tracking’ mechanisms, and the active involvement of general practitioners and social care staff. While increasing evidence supports the benefits of transitional care for older adults transitioning from hospital to home, limited literature exists on international programs, and scant evidence is available within Canada. In a recent study, in a systematic review by Barber et al. [22], the transitional care programs across Canada are identified, including the characteristics and outcomes of these programs.

Most studies in this area focus on identifying the features related to delayed discharge patients through multivariate statistical analysis [6, 7, 10, 14, 17], and investigating the costs and impacts of delayed discharge [16, 23, 24]. Additionally, few studies employ improvement methods to address the issues of ALC patients, such as implementing improvement policies for reducing delayed discharge [25, 26], and predicting delayed discharge with logistic regression models [27].

Recently, studies have begun exploring ML-based prediction models as a novel approach, using historical data to identify patients at risk of delayed discharge. For example, Ghazalbash et al. [28] suggest using ML to predict delayed discharges, showing early potential proactively. However, ML research remains primarily focused on specific patient groups (e.g., those with certain diagnoses), with limited application of predictive modeling for early risk identification across broader patient populations.

This gap in the literature indicates a need for predictive tools that consider patient-specific and systemic factors to generate actionable insights that can be applied in real-time decision-making. Addressing this need, the following section reviews studies using ML models to predict various aspects of discharge planning, highlighting their potential to improve hospital operations and patient outcomes.

### ML-based predictions in discharge planning

Research studies have recently explored various ML models to predict several aspects of patient discharges. The primary focus areas have included discharge destination, LOS, discharge time, and discharge volume. These predictive insights offer great tools for hospitals and healthcare providers to enhance the efficient use of beds, optimize staffing resources, and improve the coordination of patient care [29, 30]. However, most existing studies focus on predicting aspects of discharge for specific patient groups rather than developing comprehensive models that are applicable across diverse patient populations.

Research using historical data and ML to predict discharge destinations [31–34], discharge timing [35, 36], and LOS [37, 38]. For instance, Elbattah et al. [39] use ML models to predict LOS and discharge destinations for patients with hip fractures. They found that compared to other models, RF offers significantly higher accuracy. Bertsimas et al. [40] employed ML models to predict different aspects of patient flows, including short-term discharges, long-stay patients, and discharge destinations. Their findings demonstrate that combining patient data with interpretable ML models offers visibility into patient flows.

Furthermore, advances in interpretable ML models, such as a clinically interpretable feedforward neural network by Safavi et al. [41] and an ANN-based multi-task learning model for predicting patient LOS and inpatient flow by He et al. [42], demonstrate improved prediction accuracy. However, while RF and NN models remain top-performing techniques across different studies, few efforts have been made to test their applicability for different patient populations or broader applications [32, 43–48].

Studies like those by Ghazalbash et al. [28] and Ahn et al. [49] show potential for ML models to manage patient discharge proactively. However, most ML research focuses on specific patient groups, overlooking the integration of broader systemic and patient-specific factors that influence delayed discharge. For example, while Ghazalbash et al. effectively use ML to predict multimorbidity and resource needs, applying these insights in real-time for patient discharge remains largely unexplored.

RF, Neural Network (NN)-based models, and Gradient Boosting (GB)-based models are the most frequently used ML models in these prediction studies. RF emerged as the top-performing model across various studies [32, 35, 44, 45, 28, 46–48, 50]. Moreover, NN models showed superior performance in several investigations [34, 42, 51–53], while GB models demonstrated great performance in other studies [34, 36, 49, 54, 55].

The Literature review section presents research on delayed discharge and ALC patients and predicts discharge-related variables using ML. However, only a few studies take a holistic approach, simultaneously considering delayed discharge and predicting the factors for such patients. Research on ALC patients has mostly focused on identifying patient-related features rather than developing predictions for practice. Also, one common issue in ML studies is that they often concentrate on specific patient groups with specific conditions, neglecting the challenges of predicting discharge outcomes for patients with various medical issues, particularly those at risk of delayed discharge.

Moreover, there is a notable gap in exploring the implementation of advanced analytics to translate ML findings into actionable insights, enabling staff to improve operations. This study addresses these gaps by predicting ALC patients early and providing a practical decision tool for hospital staff.

This study addresses identified gaps by presenting an integrated predictive tool that identifies factors contributing to ALC patients and supports practical, early-stage interventions. Unlike previous research, which often limits discharge predictions to specific patient groups, this study takes a holistic approach by considering a broad range of variables, enhancing the tool’s applicability for diverse clinical settings. Additionally, it introduces a user-friendly decision-making tool that makes ML predictions directly actionable for hospital staff.

## Methodology

**Fig. 1:**
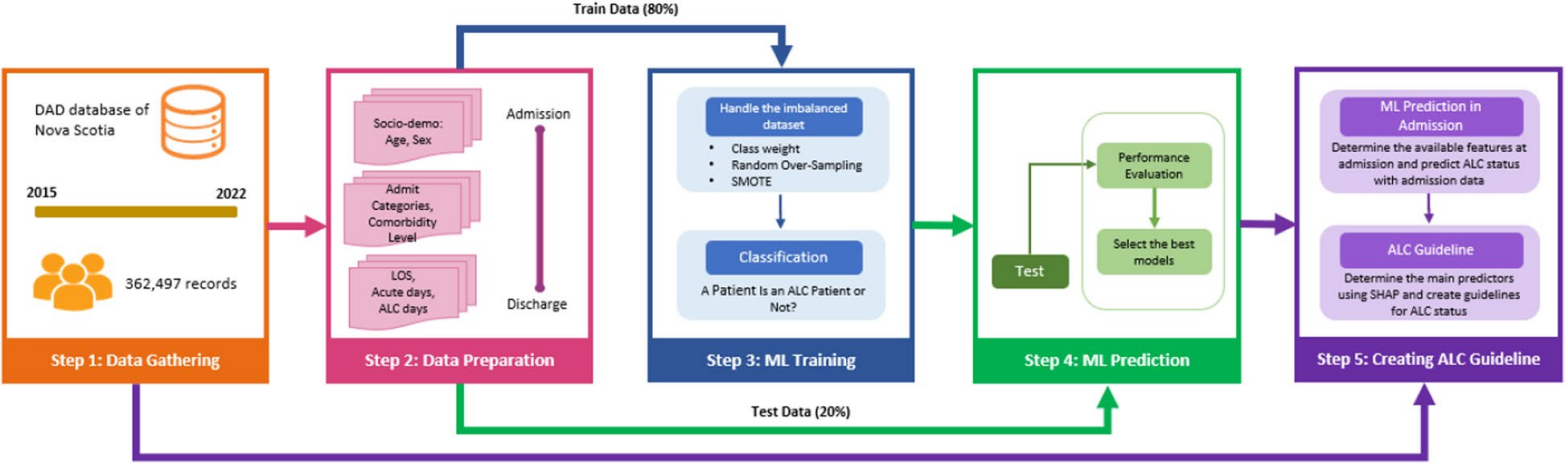
The methodological framework used in this study

This section details the methodologies employed in this study to predict ALC patients. The flow of the methodology is illustrated in Fig. 1. It begins with dataset gathering and preparation, followed by ML training, deployment, and model performance evaluation. Key predictors are then identified, forming the basis for developing an ALC prediction guideline.

### Dataset description

Data from 2015 to 2022 were extracted from the DAD of Nova Scotia Health (NSH). DAD is a Canadian national database containing information on hospital discharges, including patient demographics, diagnoses, procedures, and other relevant clinical information [56]. This dataset is used in this study given its comprehensive overview of hospital activity and patient demographics.

The features available in the dataset, along with their descriptions, are shown in Table 1. These features include patients’ anonymized Health Card Number (HCN), their demographic characteristics, admission and discharge dates, diagnostic information, admission categories, and the duration of LOS in hospitals. Table 2 provides an overview, highlighting the dataset’s characteristics and comparing ALC patients’ characteristics to the overall patient characteristics.

**Table 1:** Dataset dictionary; definitions are based on DAD data elements, CIHI; 2021 [56]

Title	Explanations
Institution number	A code assigned to a reporting facility by a provincial ministry of health identifying the facility and the level of care of the data submitted.
De-identified HCN	Health Card Number.
Admission Date	Date of admission.
Discharge Date	Date of discharge.
Readmission Code	Provides information about the patient’s previous acute care admission or day surgery visit at the reporting facility, including 1 to 9.
Patient age	Patient’s age at the time of admission.
Sex	Including two values: Female and Male.
Diagnosis	It describes the patient’s diagnosis or circumstance and is recorded using the ICD-10-CA classification code. This field may contain a single value or multiple diagnoses for some patients, with diagnosis 1 representing the primary diagnosis, followed by diagnosis 2, and so on.
Intervention Code	A code describing the services performed for or on behalf of the patient.
Admit Category	The patient’s initial status at admission to the reporting facility, including Elective, Newborn, Stillborn, and Emergent.
Entry Code	Indicates the last entry point before being admitted as an inpatient, including Clinic, Direct, Emergency department, Day surgery, and Newborn.
Transfusion Given	Whether the patient is given a transfusion, including two values: Yes and No.
CMG	A Case Mix Group is a numbered group to which an acute care inpatient is assigned, categorizing acute care patients into groups based on similarities of diagnosis and/or interventions, LOS, and resource utilization.
Main Patient Service	Describes a group of similar patients with related diagnoses, conditions, problems, or circumstances and interventions.
Comorbidity Level	The comorbidity level including six values: No significant comorbidity demonstrated by 0, Level 1, Level 2, Level 3, Level 4, Not applicable (no data for that) demonstrated by 8.
Case Weight	A numerical value assigned to each patient discharge record based on various factors such as the patient’s diagnosis, procedures performed, and other clinical characteristics.
Acute LOS	Number of days that a patient stays at the hospital for necessary treatment or service.
ALC LOS	Number of days a patient occupies an acute care bed after completing acute care treatment.

**Table 2:** Overview of dataset

Characteristics	All Patients (%)	ALC Patients (%)
	N= 362,497 (100%)	N= 17,357 (4.7%)
**Age**		
0 to 30	72,499 (20%)	226 (1.3%)
31 to 55	65,250 (18%)	660 (3.8%)
56 to 80	159,499 (44%)	7,411 (42.7%)
81+	65,249 (18%)	9,060 (52.2%)
**Sex**		
Female	198,286 (54.7%)	9,685 (55.8%)
Male	164,211 (45.3%)	7,672 (44.2%)
**ALC LOS**		
0 (non-ALC)	345,097 (95.2%)	0
1 to 10 days	4,711 (1.3%)	4,600 (26.5%)
11 to 50 days	7,613 (2.1%)	7,689 (44.3%)
51+	5,076 (1.4%)	5,068 (29.2%)
**Acute LOS**		
0 to 10 days	287,795 (79.5%)	8,279 (47.7%)
11 to 20 days	36,501 (10%)	3,298 (19%)
21 to 100 days	37,111 (10.2%)	5,433 (31.3%)
101+	1,087 (0.3%)	347 (2%)
**Total LOS**		
0 to 10 days	279,123 (77%)	2,221 (12.8%)
11 to 40 days	62,712 (17.3%)	7,724 (44.5%)
41 to 100 days	13,775 (3.8%)	5,554 (32%)
101+	6,887 (1.9%)	1,858 (10.7%)
**Entry Code**		
Clinic	16,312 (4.5%)	191 (1.1%)
Direct	109,111 (30.1%)	4,912 (28.3%)
Emergency Department	204,086 (56.3%)	12,219 (70.4%)
Day Surgery	5,075 (1.4%)	21 (0.1%)
Newborn	27,913 (7.7%)	14 (0.1%)
**Comorbidity Level**		
No significant comorbidity	239,248 (66%)	7,642 (44%)
Level 1 comorbidity	43,499 (12%)	1,811 (10.5%)
Level 2 comorbidity	38,787 (10.7%)	3,009 (17.3%)
Level 3 comorbidity	25,376 (7%)	2,951 (17%)
Level 4 comorbidity	15,587 (4.3%)	1,944 (11.2%)
**Number of Diagnoses**		
1 to 3	203,360 (56.1%)	2,829 (16.3%)
4 to 7	121,074 (33.4%)	8,435 (48.6%)
8 to 10	24,287 (6.7%)	3,315 (19.1%)
11 +	13,776 (3.8%)	2,778 (16%)

As can be seen, most patients fall within the range of 56 to 80 years (44%), followed by 0 to 35 years (20%). Conversely, most ALC patients are over 81 years old (52%). There are no differences between genders. Regarding the total LOS, 77% of patients spend less than 10 days in the hospital, whereas 42% of ALC patients stay there for more than 40 days. This emphasizes that although only 5% of patients are ALC, most stay more than 40 days in the hospital. Compared to acute LOS, data shows that 79% of all patients spend less than 10 days in the hospital, while only 47% of ALC patients do so. Conversely, 10% of patients spend 21 to 100 days in acute care, whereas this proportion for ALC patients is 31%. This indicates that ALC patients have a longer total LOS due to the additional ALC LOS portion and spend more days in acute care.

The entry code indicates that most patients are from the emergency department (53%), followed by direct (30%). Also, most ALC patients (70%) fall into the emergency department category, with direct admissions accounting for 28%. Additionally, an investigation of comorbidities reveals that ALC patients have more comorbidities than the overall patient population. Among the patient population, 66% have no significant comorbidities, 12% have level 1 comorbidities, 10% have level 2 comorbidities, 7% have level 3 comorbidities, and 4% have level 4 comorbidities. For ALC patients, the distribution is different: 44% have no significant comorbidities, 10% have level 1 comorbidities, 17% have level 2 comorbidities, 17% have level 3 comorbidities, and 11% have level 4 comorbidities.

Moreover, considering the count of diagnoses, it is observed that 56% of all patients have 1 to 3 diagnoses, followed by 33% who have 4 to 7 diagnoses. However, only 16% of ALC patients have 3 or fewer diagnoses, while the majority have 4 to 8 diagnoses (48%) and 8 to 10 diagnoses (19%). The detailed characteristics of each group of patients are highlighted in Table 2.

### Data preparation steps

Several steps are taken to prepare the data for analysis and modeling. These methods include handling outliers, engineering new features, handling missing values, transforming categorical variables, and standardizing the variables.

To identify outliers, which represent data points considered errors, visualization techniques such as scatter plots and box plots are employed [57]. The identified outliers include records where ages fall below 0 or exceed 110 years, records with ALC days exceeding 500 days, and records with acute days exceeding 200 days. Upon detection, the outliers are removed from the dataset, totaling 247 instances, which is less than 0.1% of the overall data. This approach addresses extreme values and maintains the overall central tendency of the dataset, ensuring that the overall characteristics and trends of the data remain representative and accurate [58].

Missing values are identified in several columns, including diagnosis, main patient service, CMG, transfusion given, case weight, readmission code, gender, and comorbidity level. Among these columns, 870 records had missing values, accounting for less than 0.3% of the dataset. To prevent any distortion in the analyses, these records are removed. Removing such a small fraction of data is considered a standard preprocessing step and helps maintain the accuracy of the dataset by minimizing the influence of incomplete data points on subsequent analyses [59, 60].

New features, such as “Total LOS” and “Number of Diagnoses”, are derived from some existing features. Total LOS is derived by subtracting the admission date from the discharge date. The number of diagnoses is the count of diagnoses identified for each patient. Within the dataset is a feature called ALC days, which represents the LOS for a patient while designated as ALC. Considering this feature, ALC patients are identified through a binary feature column containing a value of “1" for ALC patients and “0" for non-ALC patients.

Categorical variables are transformed into numerical representations by applying one-hot encoding for further processing. One-hot encoding represents each category within a categorical variable as a binary vector. If a data point belongs to a particular category, the corresponding column is set to 1; otherwise, it is set to 0 [61]. This process allows the algorithm to interpret and analyze categorical data as numerical values, enabling it to use this information to make predictions accurately.

Moreover, a standard scaler is utilized to compare features across the dataset. This scaler normalizes each continuous feature by subtracting the mean of the feature from each data point and then dividing it by the standard deviation of that feature [62]. Standardizing features involves bringing the data to a common scale with an average of 0 and a spread of 1.

Before using ML models, splitting the dataset into training and testing subsets is essential. To facilitate model evaluation and validation, the dataset is randomly divided into two sets: a training set comprising 80% of the data and a test set containing 20%. This step can assess the performance of predictive models and algorithms.

Additionally, addressing any imbalance issue within the dataset is crucial. An imbalance occurs when one class in the data is significantly underrepresented compared to others, potentially impacting the model’s accuracy and effectiveness. For instance, in this dataset, only 5% of patients are labeled as ALC, with the majority, 95%, classified as non-ALC. By handling this imbalance, the model can learn more effectively and make better predictions. This study explores three methods to address data imbalance: class weights, random oversampling (ROS), and employing the Synthetic Minority Over-Sampling Technique (SMOTE), which is explained in the Prediction of ALC status section.

It should be noted that all models in this study are trained using stratified 10-fold cross-validation. This involves training and evaluating the models 10 times, each using a different fold as the test set and the remaining nine as the training set. This approach helps prevent overfitting by providing multiple evaluations on different subsets of the data. Additionally, hyperparameter tuning is conducted using the grid search method [63, 64]. By tuning hyperparameters, the model can achieve improved generalization and performance. The configuration of the machine is described in Appendix A.

### Prediction of ALC status

Since predicting which patients will be ALC is a binary classification problem, three ML classifier models are tested: RF, ANN, and XGB. RF and ANN are widely acknowledged and employed in the literature for similar classification tasks [35, 38, 46]. In addition to these well-established models, XGB is also incorporated, which has shown promising performance despite being relatively novel and less used in studies [28, 49, 65]. Details on the configuration of the ANN model can be found in Appendix B.

To address the imbalanced dataset, the first approach is the application of class weight during model training. This strategy assigns different weights to the classes, with higher weights assigned to the minority class and lower weights to the majority class [66, 67]. The second approach is ROS. It is random oversampling of the minority class, which entails increasing the number of instances in the minority class by duplicating existing instances or synthesizing new ones [68]. The third approach is SMOTE, a more complex method of oversampling. SMOTE generates synthetic instances by interpolating between existing minority class instances [69, 70].

However, while these approaches, especially SMOTE, are effective for class balancing, they also carry a potential risk of overfitting, as synthetic samples may not fully capture the complexity of real-world data [71]. Overfitting can lead to exaggerated performance metrics and reduce generalizability. To mitigate this risk, stratified 5-fold cross-validation is used to assess model performance for different data splits, ensuring that results remain robust and generalizable. These steps help ensure that synthetic data does not impact the model’s ability to generalize to unseen data.

The model output represents whether the patient is categorized as ALC or not. Input features are selected using a combination of correlation matrix analysis, forward selection, and backward elimination [72–74], as integrating these methods is known to improve feature representation and support predictive modeling [75]. Correlation matrix analysis helps detect relationships between variables; forward selection gradually adds the most predictive features and backward elimination removes features that contribute less to model performance. However, this approach has limitations, such as the potential for correlation analysis to miss non-linear relationships. Additionally, forward selection and backward elimination rely on iterative processes that might be influenced by initial feature choices and model structure, potentially introducing biases if certain features are preferred over others due to sample-specific variations [74].

Considering these methods, the selected features include patient age, sex, main patient service, CMG, comorbidity level, case weight, number of diagnoses, intervention code, diagnosis 1, nursing unit, readmission code, transfusion given, admit category, and entry code. It is important to note that the DAD dataset is completed after the discharge of patients, meaning some features are available at the time of admission while others are completed post-discharge. At this stage, the goal is to predict ALC status and identify the main predictors for this objective. Therefore, the number of features is not restricted; instead, a broader set of features is considered for predicting ALC status.

Following the training phase, the models undergo testing, using 20% of the remaining data. In this step, the ML models use the inputs from the test data and try to predict the target variable “ALC status”. Next, performance evaluation metrics are used to assess the models’ performance. These metrics include precision, recall, F1-score, AUC, and specificity.

A confusion matrix is used to understand the performance metrics better, breaking down the model’s predictions into four categories. True positives represent instances that are positive and accurately predicted as positive by the model. True negatives denote negative instances correctly predicted as negative by the model. False positives account for instances that are negative but are incorrectly predicted as positive by the model, constituting type I errors. Conversely, false negatives encompass instances that are positive but are mistakenly predicted as negative by the model, illustrating type II errors.

Recall or sensitivity measures the model’s ability to correctly identify positive instances, while precision provides insight into its ability to avoid false positives. A higher recall indicates fewer false negatives, while a high precision value indicates that the model makes accurate positive predictions with few false positives. Moreover, Specificity assesses the model’s ability to correctly identify negative instances, indicating its reliability in avoiding false positives.

F1-score captures the trade-off between false negatives and false positives. A higher F1-score indicates a better balance between precision and recall as it combines precision and recall into a single metric using this formula:

$$F1 = 2 \times \frac{precision \times recall}{precision + recall}$$

AUC is another metric used to evaluate the performance. AUC calculates the area under the Receiver Operating Characteristic Curve (ROC), which plots the true positive rate against the false positive rate at different classification thresholds. A perfect model would have an AUC value of 1, indicating perfect discrimination between ALC and non-ALC. In contrast, a model with no discrimination ability would have an AUC value of 0.5, equivalent to random guessing.

## Results

This section presents the outcomes of the ML models used to classify ALC patients. First, the full-feature models are assessed to identify the main predictors of ALC status. Next, the feasibility of predicting ALC status using only admission data is evaluated, leading to a streamlined model with three key features.

### Full features model

This section presents the results of ML models in classifying ALC patients. In predicting ALC status, misclassifying ALC patients as non-ALC is more detrimental than misclassifying non-ALC patients as ALC. Therefore, focusing on recall and AUC is more appropriate to minimize false negatives and ensure that ALC patients are correctly identified.

Table 3 shows the performance of RF, ANN, and XGB models in predicting ALC patients (class 1). As shown in Table 3, the RF model combined with the SMOTE approach achieves the best recall and AUC compared to RF with other approaches. The combination with the class weight approach outperforms the other methods for the ANN model, resulting in the highest recall and AUC. Lastly, the XGB model, when combined with SMOTE, excels in recall and AUC and outperforms other models across all five metrics, showing its effectiveness in capturing true positives while minimizing false negatives. Following the XGB model with SMOTE, the next best-performing models are the XGB with ROS, and the ANN combined with the class weight. Also, the performance metrics results, along with 95% confidence intervals, are presented in Table 7 in Appendix C to provide a robust assessment of the model’s reliability.

**Table 3:** Performance of the three ML models in predicting ALC patients considering full features

ML Models	Balance Approaches	Precision	Recall	F1-Score	AUC	Specificity
**RF**	Class Weight	0.82	0.57	0.67	0.77	0.87
	ROS	0.75	0.66	0.70	0.82	0.93
	SMOTE	0.59	0.76	0.66	0.86	0.82
**ANN**	Class Weight	0.37	0.84	0.51	0.85	0.74
	ROS	0.46	0.81	0.58	0.81	0.83
	SMOTE	0.44	0.52	0.47	0.73	0.79
**XGB**	Class Weight	0.98	0.70	0.82	0.85	0.91
	ROS	0.54	0.92	0.68	0.86	0.85
	SMOTE	**0.87**	**0.95**	**0.91**	**0.97**	**0.93**

To ensure the XGB model with SMOTE is not overfitting, a stratified 5-fold cross-validation approach is applied, calculating the mean and standard deviation of performance metrics across all folds, as shown in Table 10 in Appendix D. Consistent values across folds indicate that the model generalizes well and does not overfit to any specific data subset.

**Fig. 2:**
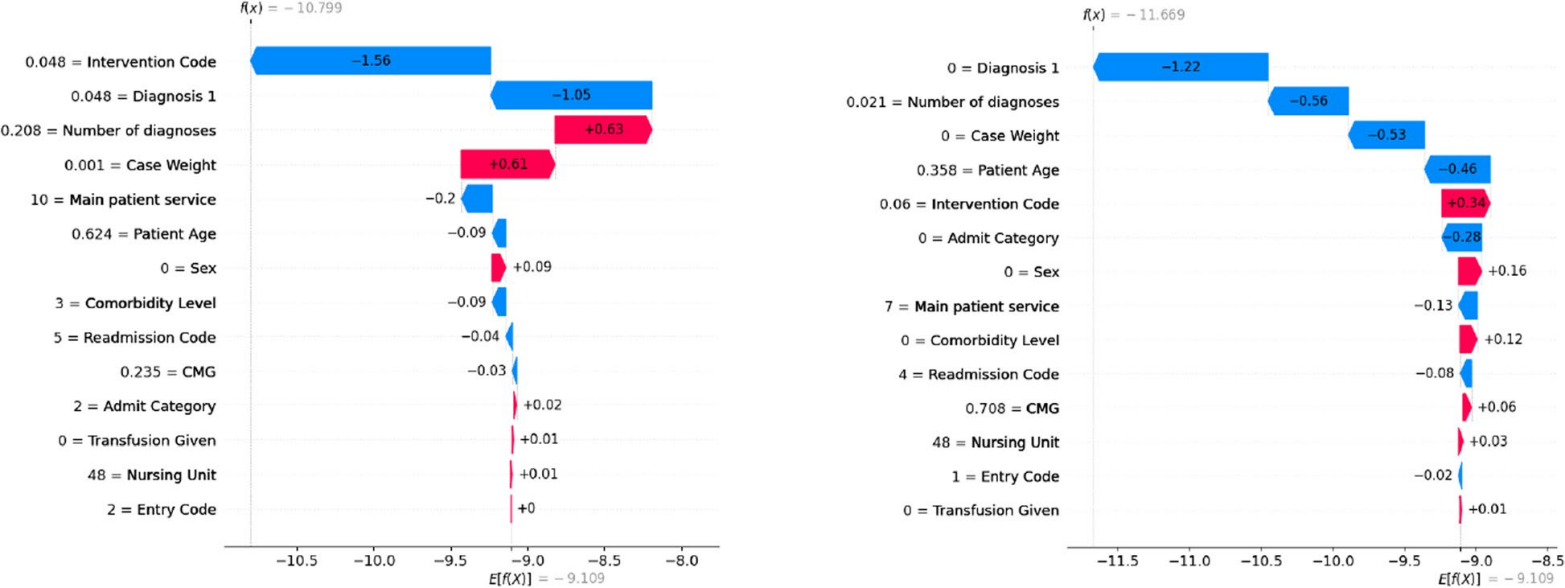
SHAP values for two specific instances within the XGB classifier for predicting ALC patients considering full features

As the XGB model can accurately predict the ALC status of patients, it is now important to identify which features are the main predictors of ALC status. SHAP analyses are conducted to determine the significant predictors. SHAP values are a widely used technique for understanding the contribution of each feature to a model’s prediction [76, 77]. Figure 2 shows waterfall plots for two patient instances. On the left, a non-ALC patient is predicted as non-ALC by the model (true negative), and on the right, an ALC patient is predicted as ALC (true positive). The numbers inside the red (blue) arrows indicate the input variables influencing the ML model to predict a patient as ALC (non-ALC). For non-ALC patients, the intervention code, diagnosis 1, and the number of diagnoses are the most important factors in prediction. For ALC patients, diagnosis 1, number of diagnoses, and case weight are the most important factors in prediction, respectively.

**Fig. 3:**
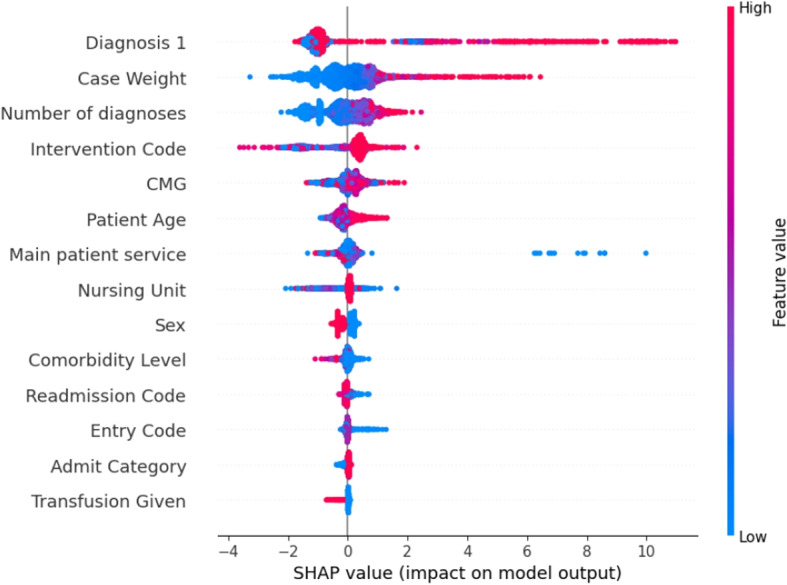
SHAP values illustrating feature contributions within the XGB classifier for predicting ALC patients considering full features

Additionally, Fig. 3 presents a bee swarm plot, a visualization method that highlights how the critical features in the dataset, at an aggregate level, affect whether a patient is classified as ALC. Figure 3 shows that the top predictors of ALC status in the XGB model are diagnosis 1, case weight, number of diagnoses, intervention code, CMG, patient age, and main patient service. It should be noted that the red color (value 1) indicates that a feature had an impact on labeling a patient as ALC (class 1), while the blue color (value 0) signifies that the patient is labeled as non-ALC (class 0). The purple colors represent values between 0 and 1. This analysis indicates that these features are crucial for accurately predicting ALC status. However, some of these features are only available at the time of discharge. To determine if it is possible to accurately predict ALC status using data available upon admission, only the features accessible at that time are considered in an additional application of the model described in Admission features model section. This will assess whether ALC status can be accurately predicted at admission or at any point during the patient’s hospital stay before discharge.

### Admission features model

This section evaluates the effectiveness of ML models in identifying ALC patients using features available at the time of admission. While previous results show that ML models can accurately predict ALC status using all dataset features, predicting it at admission is more challenging because many of the features will become available during the hospital stay and are unavailable at admission.

The available features at the time of admission include age, sex, readmission code, diagnosis 1, entry code, and admit category. Although this selection may slightly reduce the accuracy of some ML models compared to previous results, it enables the hospital staff to predict ALC status sooner.

Table 4 shows the performance of models based on admission features. Notably, models using the admission features exhibit lower accuracy than those with the initial feature set. RF’s performance decreases, with its best to be with class weight, achieving a recall of 0.69 and AUC of 0.71. The top-performing ANN model is noted with the ROS method, presenting a recall of 0.83 and an AUC of 0.89. However, XGB with the SMOTE maintains strong performance, with a recall of 0.91 and AUC of 0.94, indicating its ability to predict ALC patient status upon admission accurately. The 95% confidence intervals for the performance metrics are presented in Table 8 in Appendix C. Also, stratified 5-fold cross-validation was used (see Table 11 in Appendix D) to confirm that the XGB model with SMOTE is not overfitting.

**Table 4:** Performance of the three ML models in predicting ALC patients, considering admission features

ML Models	Balance Approaches	Precision	Recall	F1-Score	AUC	Specificity
**RF**	Class Weight	0.21	0.69	0.32	0.71	0.57
	ROS	0.22	0.66	0.33	0.72	0.58
	SMOTE	0.25	0.66	0.36	0.72	0.62
**ANN**	Class Weight	0.19	0.78	0.30	0.85	0.54
	ROS	0.28	0.83	0.42	0.89	0.66
	SMOTE	0.13	0.56	0.21	0.79	0.42
**XGB**	Class Weight	0.76	0.42	0.54	0.61	0.94
	ROS	0.63	0.89	0.74	0.90	0.89
	SMOTE	0.71	**0.93**	0.80	**0.94**	0.92

**Fig. 4:**
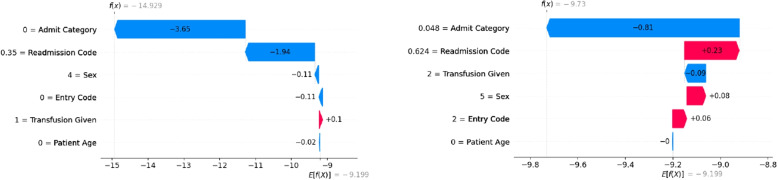
SHAP values for two specific instances within the XGB classifier for predicting ALC patients considering admission features

To determine the significant predictors upon admission, SHAP analyses are conducted to help understand the impact of features on model predictions. Figure 4 shows waterfall plots for two patient instances. On the left, the model predicts a non-ALC patient as non-ALC, and on the right, an ALC patient is predicted as ALC. It can be observed that for both patients, diagnosis 1 and patient age are the most important factors in prediction. Furthermore, while the admit category is the third most important factor in predicting non-ALC patients, the entry code is the third most important factor in predicting ALC patients.

**Fig. 5:**
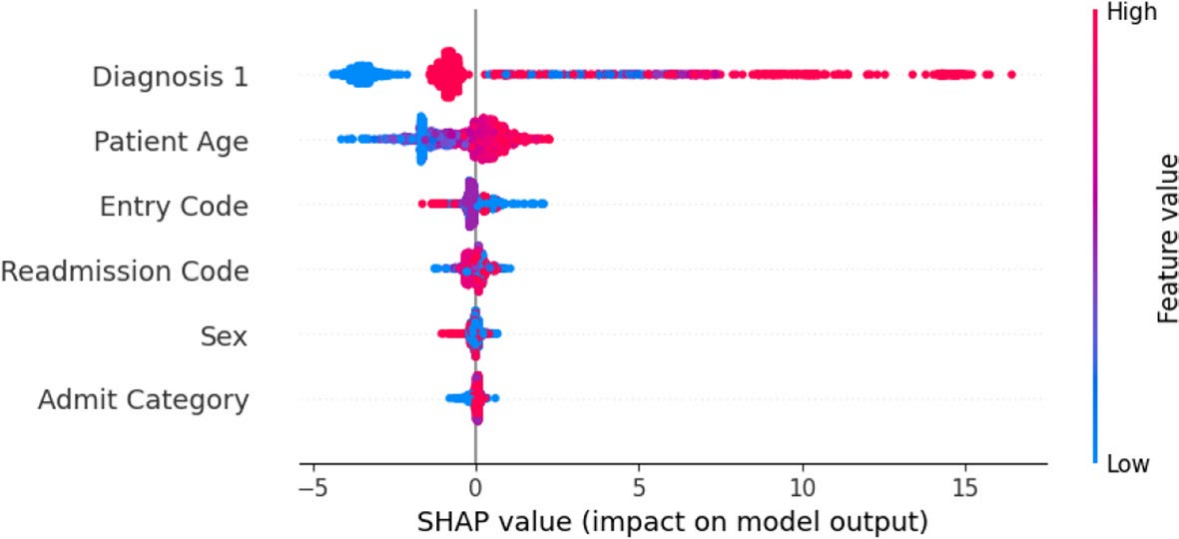
SHAP values illustrating feature contributions within the XGB classifier for predicting ALC patients considering admission features

Additionally, as shown in the bee swarm plot in Fig. 5, diagnosis 1 is the most crucial feature, followed by patient age and entry code.

### Creating a guideline to predict ALC with limited features

While the ML model represents its ability to accurately identify ALC patients upon admission, its complexity may present challenges for hospital staff, limiting its practicality. To simplify the prediction, a streamlined model with three features is tested, and a policy is developed to predict the classification of ALC patients at the time of admission. As discussed, the most influential factors identified through SHAP analysis are diagnosis 1, patient age, and entry code. Considering these three features, the XGB model is employed to evaluate the performance.

Table 5 presents the performance of this limited-feature model in predicting ALC patients at the time of admission. As expected, performance decreased, but the recall remained sufficiently high for practical application. In practice, a recall of 0.91 means the model correctly identifies 91% of true positive cases, ensuring that most relevant cases are captured for effective decision-making. This indicates that while the limited-feature model may miss some ALC patients with more complex needs, it remains effective and easier for hospital staff to implement in real-time. The 95% confidence intervals for the performance metrics are presented in Table 9 in Appendix C. To confirm that the XGB model with SMOTE is not overfitting, stratified 5-fold cross-validation was used, as shown in Table 12 in Appendix D.

**Table 5:** Performance of the XGB ML model in predicting ALC patients, considering only the top three admission features

ML Models	Balance Approaches	Precision	Recall	F1-Score	AUC	Specificity
**XGB**	SMOTE	0.64	0.91	0.75	0.82	0.87

The guideline to predict ALC status is created based on a range of feature values representing typical patients. The first feature, diagnosis 1, is initially classified into eleven disease groups based on the ICD-10 coding system [78]. In this section, the minor categories are integrated to streamline the information and reduce the number of tables, resulting in six overarching categories as shown in Table 6. The categories are: 1. Neurological diseases, eye diseases, and mental disorders; 2. Pulmonary diseases; 3. Cardiac and circulatory diseases; 4. Gastrointestinal diseases and genital/urinary diseases; 5. Metabolic diseases, blood, and systemic diseases; and 6. Musculoskeletal diseases.

The categories of entry codes used in the guideline are shown in Table 6, including Clinic, Direct, Emergency department, and Day surgery.

**Table 6:** Feature categories of sample patients

Feature	Category
**Diagnosis 1**	Neurological Diseases, Eye Diseases, and Mental Disorders
	Pulmonary Diseases
	Cardiac and Circulatory Diseases
	Gastrointestinal Diseases, Genito/Urinary Diseases
	Metabolic Diseases, Blood, and Systemic Diseases
	Musculoskeletal Diseases
**Entry Code**	Clinic
	Direct
	Emergency Department
	Day Surgery
**Patient age**	0–36
	37–63
	64–76
	77–108

Analyzing the patient’s age through a histogram, it is found that the minimum count is 0, the first quartile (Q1) is 37, the second quartile (Median) is 64, the third quartile (Q3) is 77, and the maximum is 108. Based on this analysis, this feature is classified into four groups (0–36, 37–63, 64–76, 77–108).

All possible combinations of patient features are generated from the features highlighted in Table 6. For example, a subset of sample patients with a “diagnosis 1” of “pulmonary diseases” would include patients with all values of “patient age” ranging from 0 to 108 while keeping all other features constant. This process is repeated for every value of every feature.

**Fig. 6:**
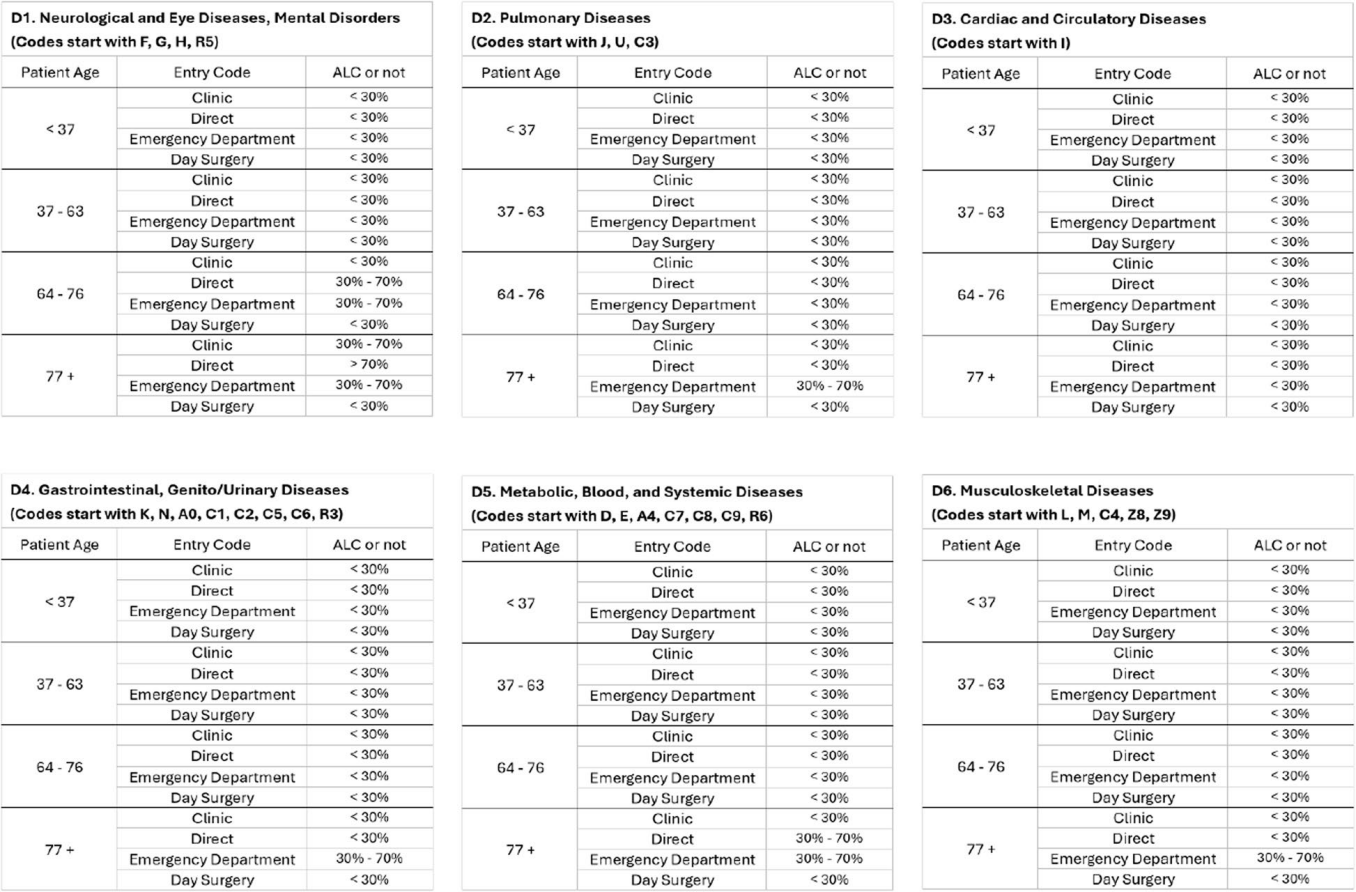
ALC probability classification guideline

Based on the top three predictors of ALC status, the ALC guideline is outlined and illustrated in Fig. 6. Patients are first grouped by diagnosis 1. Within each diagnosis 1 group, patients are divided into four distinct groups by age: under 37, 37 to 63, 64 to 76, and 77 or older. These groups are then further divided based on the entry code: under clinic, direct, emergency department, and day surgery. The prediction outcomes are categorized into three probabilities: less than 30%, between 30% and 70%, and greater than 70%. For example, a patient admitted with a primary diagnosis of pulmonary diseases, age over 77 years, and entry code of the emergency department has a 30% to 70% likelihood of being classified as ALC. The necessary data to apply these guidelines are easily accessible to staff, allowing the guide to be used directly without software development.

## Conclusion

Early prediction of ALC patients supports proactive discharge planning by alerting staff sooner. Knowing which patient will be ALC in advance enables better resource planning and allocation, ultimately reducing ALC-related bottlenecks and improving hospital patient flow.

This study investigates the application of ML models to predict ALC patients by analyzing features of patients, including demographic information and medical conditions. It addresses three fundamental objectives: identifying individuals likely to be classified as ALC patients, the features influencing these predictions, and creating guidelines to determine ALC patients at the time of admission.

In the first phase, a binary classification task is conducted using the full features, employing three distinct ML models: RF, ANN, and XGB. The performance of the models is assessed using recall to evaluate the effectiveness of the classifier in identifying the ALC patients, F1-score to combine the values of recall and precision through harmonic means, specificity to measure the model’s ability to identify non-ALC cases correctly, and AUC to evaluate the model’s ability to discriminate between ALC and non-ALC patients. The performance evaluation reveals the superiority of the XGB model using SMOTE in predicting ALC patients, highlighting its effectiveness in handling imbalanced datasets.

Next, the main predictors of ALC status are determined using SHAP analysis. Based on this analysis, the features included in the model were restricted to those available at the time of admission. As expected, the performance of the model diminishes, but recall remains above 0.9, demonstrating the practicality of the model.

Finally, a more streamlined model is tested to facilitate implementation. This model incorporates only the three most influential features. Although the overall performance of this limited-feature model decreases, the recall rate remains above 0.9. By using just these three features, the likelihood of ALC for typical values of these features can be predicted. The guideline is designed to be widely applicable because it is based on standard patient characteristics rather than region-specific factors. By relying on these patient features, it can be implemented in various settings where standard coding practices are in use.

However, a trade-off exists between model complexity and usability in practical settings. While a full-feature ML model may offer higher accuracy, its complexity can limit practicality for hospital staff due to the need for extensive data inputs and complex interpretation. In contrast, despite having lower overall performance, the limited-feature model provides a clear advantage in simplicity. Its predictions can be summarized in an easy-to-read guide, making it accessible for healthcare professionals without requiring technical expertise or advanced systems.

With a recall of 0.91, the limited-features model effectively identifies most ALC cases, balancing precision and accessibility. While it may miss some patients with complex needs, it remains a reliable tool for real-time use, supporting early ALC identification and timely decision-making in resource-limited settings. This balance of simplicity and accuracy highlights the model’s potential for practical application, enabling improved patient care and resource allocation.

In addition to identifying patients at risk of becoming ALC, this predictive model has practical applications for hospital operations and patient care. Early identification of ALC patients allows hospitals to proactively engage discharge planning teams, coordinate with LTC facilities, and prepare necessary social and community services, thus minimizing discharge delays. By predicting ALC status, hospitals can allocate resources more effectively, ensuring that acute care beds are available for patients in need.

Furthermore, understanding patients’ potential next destinations, such as LTC facilities or other community-based resources, enhances care transitions and reduces system bottlenecks. This approach not only improves hospital and patient flow but also contributes to better healthcare resource management, benefiting both patients and the healthcare system.

This study uses a large, real-world dataset to uncover key factors linked to ALC patients, creating a foundation for predicting ALC status at the time of admission. These insights are used to develop a user-friendly manual guideline, making it easier to identify ALC patients early. This unique strength sets this work apart from other studies. The study translates ML findings into practical insights by applying advanced analytics, helping staff improve efficiency and enhance operations.

While this study offers valuable insights, it also has limitations that point to potential areas for future research. One limitation is the lack of specific clinical interventions and real-time data, as the dynamic nature of patients’ experiences within the hospital presents various risks that may impact prediction outcomes. Another limitation is the lack of socioeconomic features in the dataset. Socioeconomic characteristics such as marital status, urban versus rural residence, and financial status can influence the ALC status of patients, as described in the Literature review section.

The other limitation of this study is that the dataset is for a single health system. While the approach and method may be applicable elsewhere, the results and guidelines will likely differ. Focusing on implementing this model across different datasets is an opportunity for future work.

Given that ALC LOS directly impacts ALC patient outcomes and hospital congestion, incorporating the prediction of LOS into the results presents an excellent opportunity for further studies. Another area for future research involves exploring the practical applications of predictions, considering the next destination of ALC patients, including LTCs, to improve healthcare system processes and resource management.

## Figures and Tables

**Fig. 7:**
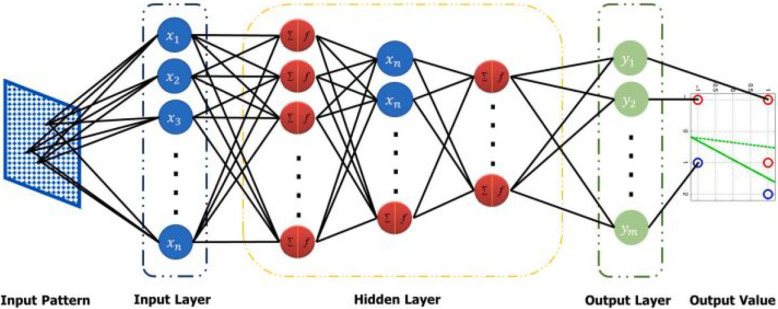
General structure of ANN in this study [79]

**Fig. 8:**
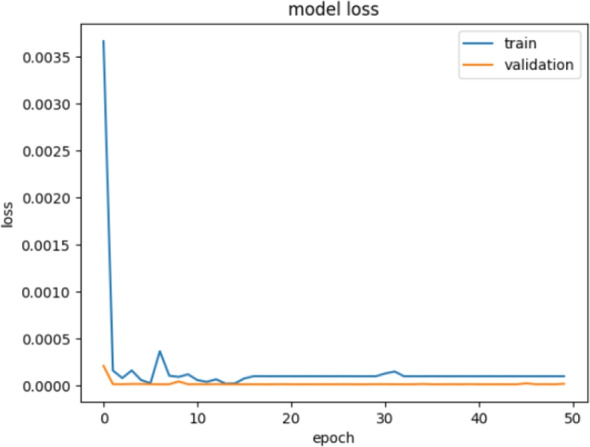
Loss curve of ANN model

## Data Availability

The data that support the findings of this study are available from Nova Scotia Health, but restrictions apply to the availability of these data, which were used under license for the current study and so are not publicly available. Data are, however available from the authors upon reasonable request and with permission of Nova Scotia Health.

## Appendix A: machine configuration

This appendix details the machine configuration used for implementing the ML models discussed in the study.

### Hardware specifications

CPU: Intel Xeon

GPU: NVIDIA Tesla T4

RAM: 12 GB

Storage: 120 GB

Operating System: Ubuntu

### Software environment

Python Version: 3.9.5

Development Environment: Google Colaboratory

Machine Learning Libraries:

scikit-learn 0.24.2

TensorFlow 2.5.0

Keras 2.4.3

XGBoost 1.4.2

### Additional notes

All ML models were implemented and trained on the specified hardware and software environment.

The NVIDIA GPU was used for deep learning tasks, leveraging CUDA acceleration provided by the TensorFlow framework. Model training and evaluation were conducted using Google Colab.

## Appendix B: ANN model configuration

### Model architecture

As shown in Fig. 7, the architecture of the ANN model consists of three main components:

**Input Layer:** The input layer receives the features extracted from the dataset, each serving as an input node to the network. Features are selected using correlation matrix analysis, forward selection, and backward elimination techniques. The selected features include patient age, sex, main patient service, CMG, comorbidity level, case weight, number of diagnoses, intervention code, diagnosis 1, nursing unit, readmission code, transfusion given, admit category, and entry code.

**Hidden Layers:** The hidden layers contain multiple neurons organized in one or more hidden layers. These neurons perform computations and introduce non-linear transformations to the input data.

**Output Layer:** The output layer produces predicted outcomes based on the input data and the learned parameters of the network. For the classification task, the output represents whether the outcome is ALC (class 1) or not (class 0).

### Hyperparameters

The model architecture comprises 8 fully connected layers, each with varying neurons per layer, capped at a maximum of 64 neurons.

Also, The activation function employed across all layers, except the final one, is the Rectified Linear Unit (ReLU), while the last layer utilizes softmax activation.

### Training procedure

The training process involved iteratively updating the model parameters (weights and biases) to minimize a predefined loss function using Adam. With a learning rate of 0.001, the Adam optimizer is utilized alongside the binary cross-entropy loss function. Figure 8 represents the loss curve of the ANN model during its training. As can be seen, the curve depicts how the loss value evolves over the training process. It starts at a relatively high loss, and then, as the model iteratively learns from the training data, the loss steadily decreases.

Furthermore, training is conducted over 50 epochs, and early stopping is implemented to mitigate overfitting risks. This comprehensive configuration integrates various components to optimize model performance and address potential overfitting concerns.

## Appendix C: performance metrics with confidence intervals for ML models

Tables 7, 8, and 9 present the performance metrics for the ML models, including confidence intervals to provide a robust measure of reliability and precision. Each metric is reported with a 95% confidence interval to account for variability across different model runs, offering a clearer view of model performance.

**Table 7:** Performance metrics with 95% confidence intervals for the three ML models in predicting ALC patients considering full features

ML Models	Balance Approaches	Precision CI	Recall CI	F1-Score CI	AUC CI	Specificity CI
**RF**	Class Weight	0.82 ± 0.0027	0.57 ± 0.0036	0.67 ± 0.0034	0.77 ± 0.0030	0.87 ± 0.0024
	ROS	0.75 ± 0.0031	0.66 ± 0.0034	0.70 ± 0.0033	0.82 ± 0.0027	0.93 ± 0.0018
	SMOTE	0.59 ± 0.0035	0.76 ± 0.0031	0.66 ± 0.0034	0.86 ± 0.0025	0.82 ± 0.0027
**ANN**	Class Weight	0.37 ± 0.0035	0.84 ± 0.0026	0.51 ± 0.0036	0.85 ± 0.0026	0.74 ± 0.0031
	ROS	0.46 ± 0.0036	0.81 ± 0.0028	0.58 ± 0.0035	0.81 ± 0.0028	0.83 ± 0.0027
	SMOTE	0.44 ± 0.0036	0.52 ± 0.0034	0.47 ± 0.0035	0.73 ± 0.0032	0.79 ± 0.0027
**XGB**	Class Weight	0.98 ± 0.0011	0.70 ± 0.0034	0.82 ± 0.0027	0.85 ± 0.0026	0.91 ± 0.0017
	ROS	0.54 ± 0.0034	0.92 ± 0.0022	0.68 ± 0.0034	0.86 ± 0.0025	0.85 ± 0.0020
	SMOTE	0.87 ± 0.0024	0.95 ± 0.0017	0.91 ± 0.0024	0.97 ± 0.0014	0.93 ± 0.0018

**Table 8:** Performance metrics with 95% confidence intervals for the three ML models in predicting ALC patients considering admission features

ML Models	Balance Approaches	Precision CI	Recall CI	F1-Score CI	AUC CI	Specificity CI
**RF**	Class Weight	0.21 ± 0.0029	0.69 ± 0.0033	0.32 ± 0.0033	0.71 ± 0.0033	0.57 ± 0.0036
	ROS	0.22 ± 0.0030	0.66 ± 0.0034	0.33 ± 0.0034	0.72 ± 0.0032	0.58 ± 0.0035
	SMOTE	0.25 ± 0.0031	0.66 ± 0.0034	0.36 ± 0.0034	0.72 ± 0.0032	0.62 ± 0.0035
**ANN**	Class Weight	0.19 ± 0.0028	0.78 ± 0.0030	0.30 ± 0.0033	0.85 ± 0.0026	0.54 ± 0.0036
	ROS	0.28 ± 0.0032	0.83 ± 0.0027	0.42 ± 0.0035	0.89 ± 0.0022	0.66 ± 0.0034
	SMOTE	0.13 ± 0.0036	0.56 ± 0.0029	0.21 ± 0.0025	0.79 ± 0.0028	0.42 ± 0.0031
**XGB**	Class Weight	0.76 ± 0.0032	0.42 ± 0.0033	0.54 ± 0.0034	0.61 ± 0.0033	0.94 ± 0.0021
	ROS	0.63 ± 0.0034	0.89 ± 0.0022	0.74 ± 0.0033	0.90 ± 0.0020	0.89 ± 0.0020
	SMOTE	0.71 ± 0.0032	0.93 ± 0.0017	0.80 ± 0.0030	0.94 ± 0.0014	0.92 ± 0.0018

**Table 9:** Performance metrics with 95% confidence intervals for the three ML models in predicting ALC patients, considering only the top three admission features

ML Models	Balance Approaches	Precision CI	Recall CI	F1-Score CI	AUC CI	Specificity CI
**XGB**	SMOTE	0.64 ± 0.0035	0.91 ± 0.0021	0.75 ± 0.0032	0.82 ± 0.0028	0.87 ± 0.0017

## Appendix D: Cross-Validation Performance Metrics to Assess Models Generalizability

To ensure the XGB model in different applications does not overfit, a stratified 5-fold cross-validation was conducted for each model, with performance metrics including Precision, Recall, F1-Score, AUC, and Specificity calculated across each fold. Table 10 summarizes these metrics for the best-performing model considering full features, Table 11 indicates metrics for the best-performing model considering admission features, and Table 12 shows performance metrics for the best-performing model (XGB with SMOTE) considering only the top three admission features.

The results show consistency across folds. Each metric’s low standard deviation values indicate stable performance across different data subsets, confirming the models’ generalizability and minimizing overfitting concerns.

**Table 10:** Performance metrics across 5-Fold Cross-Validation for the best-performing model (XGB with SMOTE) considering full features

	Precision	Recall	F1-Score	AUC	Specificity
Fold 1	0.87	0.94	0.90	0.97	0.86
Fold 2	0.95	0.89	0.92	0.98	0.95
Fold 3	0.78	0.96	0.86	0.95	0.92
Fold 4	0.89	0.93	0.91	0.97	0.89
Fold 5	0.84	0.95	0.89	0.97	0.87
Mean	0.87	0.93	0.90	0.97	0.90
Standard Deviation	0.06	0.03	0.02	0.01	0.04

**Table 11:** Performance metrics across 5-Fold Cross-Validation for the best-performing model (XGB with SMOTE) considering admission features

	Precision	Recall	F1-Score	AUC	Specificity
Fold 1	0.74	0.94	0.83	0.92	0.87
Fold 2	0.81	0.89	0.85	0.95	0.89
Fold 3	0.69	0.95	0.80	0.95	0.93
Fold 4	0.73	0.89	0.80	0.93	0.82
Fold 5	0.62	0.96	0.75	0.94	0.94
Mean	0.72	0.93	0.81	0.94	0.89
Standard Deviation	0.07	0.03	0.04	0.01	0.05

**Table 12:** Performance metrics across 5-Fold Cross-Validation for the best-performing model (XGB with SMOTE) considering only the top three admission features

	Precision	Recall	F1-Score	AUC	Specificity
Fold 1	0.72	0.81	0.76	0.84	0.83
Fold 2	0.67	0.89	0.76	0.72	0.86
Fold 3	0.59	0.95	0.73	0.88	0.88
Fold 4	0.61	0.94	0.74	0.92	0.76
Fold 5	0.58	0.94	0.72	0.80	0.92
Mean	0.63	0.91	0.75	0.83	0.85
Standard Deviation	0.06	0.06	0.02	0.08	0.06
